# Microneedle physical contact as a therapeutic for abnormal scars

**DOI:** 10.1186/s40001-017-0269-6

**Published:** 2017-08-14

**Authors:** David C. Yeo, Elizabeth R. Balmayor, Jan-Thorsten Schantz, Chenjie Xu

**Affiliations:** 1 School of Chemical and Biomedical Engineering, Nanyang Technological University, 70 Nanyang Drive, Singapore, 637457 Singapore; 2 Department of Plastic Surgery and Hand Surgery, Klinikum rechts der Isar, Technical University of Munich, Munich, Germany; 3 Institute for Advanced Study, Technical University of Munich, Munich, Germany; 4 NTU-Northwestern Institute for Nanomedicine, Nanyang Technological University, Singapore, Singapore

## Abstract

**Background:**

Abnormal (keloid and hypertrophic) scars are a significant affliction with no satisfactory single modality therapy to-date. Available options are often ineffective, painful, potentially hazardous, and require healthcare personnel involvement. Herein a self-administered microneedle device based on drug-free physical contact for inhibiting abnormal scars is reported. Its therapeutic activity through microneedle contact eliminates hazards associated with toxic anti-scarring drugs while self-treatment enables administration flexibility.

**Methods:**

The microneedle patch was fabricated with FDA-approved liquid crystalline polymer under good manufacturing practice. It was first tested to ascertain its ability to inhibit (keloid) fibroblast proliferation. Later the microneedle patch was examined on the rabbit ear hypertrophic scar model to explore its potential in inhibiting the generation of abnormal scars post-injury. Finally, the microneedle patch was applied to the caudal region of a hypertrophic scar located on a female patient’s dorsum to verify clinical efficacy.

**Results:**

On untreated control cultures, barely any non-viable fibroblasts could be seen. After 12-h treatment with the microneedle patch, the non-viable proportion increased to 83.8 ± 11.96%. In rabbit ear hypertrophic scar model, 100% of the control wounds without the presence of patches on rabbit ears generated regions of raised dermis originating from the wound site (3/3), whereas microneedle treatment prevented dermis tissue thickening in 83.33% of the wounds (15/18). In the clinical test, the microneedle patch was well tolerated by the patient. Compared to the untreated region, microneedle treatment decreased the number of infiltrated inflammatory cells, with less disrupted dermis tissue architecture and more flattened appearance.

**Conclusions:**

A self-administered, drug-free microneedle patch appears highly promising in reducing abnormal scarring as observed from in vitro, in vivo and clinical experiments. Larger cohort clinical studies need to be performed to validate its efficacy on abnormal scars.

**Electronic supplementary material:**

The online version of this article (doi:10.1186/s40001-017-0269-6) contains supplementary material, which is available to authorized users.

## Background

The occurrence of abnormal scars—keloids and hypertrophic scars persist as a significant clinical problem, especially given the high incidence of keloids in African and Hispanic populations (up to 16% [1]). While a broad range of treatment options are available, each has significant limitations and is not completely satisfactory [2–4]. Intralesional injection of triamcinolone acetonide (TAC) is the first-line option for treatment of keloids, and second-line for treatment of hypertrophic scars [5]. TAC injections are repeatedly administered for a period of 2–5 weeks at weekly intervals, although recurrence rates can vary between 9 and 50%. Other drugs such as anti-metabolites (5-fluorouracil, bleomycin etc.) and interferons are also delivered via injection, often causing significant pain [5]. While surgical excision is used for the removal of hypertrophic scars, its prescription is highly subjective since wound tension must be minimal [5]. As a single modality, surgery is not effective against keloid scars [2, 3]. Laser and radiotherapy are also promising options [2] although these are equipment-intensive and can pose malignancy risks (i.e., ionizing radiation) [6]. Contraindications further exclude therapeutic administration, particularly of toxic substances. Dressing-based therapy (e.g., silicone gel sheeting), while non-invasive, does not have strong evidence of efficacy. In addition, reported studies are often insufficiently well designed [5, 7].

Microneedles have gained popularity over the past decade primarily for transdermal drug delivery [8] and more recently for derma-abrasion usage in aesthetic medicine [9]. Previously, we have developed microneedle devices for the controlled release of growth factors [10] and 5-fluorouracil [11] utilized in tissue regeneration and dermatology applications respectively. Fortuitously, we observed that drug-free microneedles exhibit contact-dependent inhibition on keloid and normal fibroblast proliferation [11], implying that microneedle contact potentially suppresses the formation/growth of abnormal scars. Using drug-free, physical methods for therapy eliminates the reliance on potentially toxic substances (e.g., 5-fluorouracil and radiotherapy) making it a safer therapeutic approach. Microfabrication provides the flexibility to design microneedle devices of desirable parameters. For example, needles can be made sufficiently deep to penetrate the epidermis (50–81 μm [12]), yet avoid contact with the dermis region in hypertrophic scars that have increased nerve density [13]. This potentially reduces the level of pain experienced by patients with abnormal scars compared to injection-based therapeutics. Additionally, microneedles are easy to use and can be self-administered with minimal supervision, reducing the involvement of medical specialists during treatment. In this article, we introduce the usage of drug-free microneedles as a potential therapeutic for abnormal scars by demonstrating its efficacy in pre-clinical models (keloid fibroblast cell culture and a rabbit ear hypertrophic scar model) and report our findings from preliminary clinical procedures.

## Patients and methods

### Microneedle design and fabrication

Microneedles are composed of a liquid crystal polymer (LCP) Vectra^®^ MT1300 (Ticona, Celanese Corporation) which has filed drug (DMF No. 8468) and master (MAF No. 315) files with the US Food and Drug Administration (FDA). Microneedles were designed using the following parameters: needle height of 500 μm, density of 21 needles per cm (or 441 needles per cm^2^), fabricated in a 10 × 10 needle array. Individual microneedles are shaped as half-pyramids for ease of fabrication. LCP was chosen for its safety and high mechanical toughness, to withstand microneedle compression against skin. Fabrication involved an injection molding process with the following parameters: injection melt temperature of 293 °C, mold temperature between 60 and 120 °C, injection velocity of 150 mm/s and hold pressure of 48 MPa. Further details of the process can be found from the manufacturer’s insert provided by Ticona Engineering Polymers. Injection-molds consisting of stacked insert shims were inserted into a mold-set since the seams between the insert shims allow the escape of trapped air during the injection molding process (Micropoint Technologies Pte Ltd, Singapore).

### Fibroblast culture and analysis

Keloid fibroblasts (KF110) were purchased from Cell Research Corporation (Singapore). These cells were harvested during surgical excision of a keloid scar from the cheek region of a male patient of Malay ethnicity, 9 years of age. Passage 4 or 5 cells were used for cell culture experiments reported in this study. KF110 cells were cultivated using Dulbecco’s minimum essential medium (DMEM) containing antibiotics (1% penicillin–streptomycin) and fetal bovine serum (10% FBS). Normal fibroblasts (product code NF200) were also purchased from Cell Research Corporation with the cells used up to passage 10. Cell culture, microneedles treatment, and imaging steps were performed similar to the keloid fibroblasts (KF110). KFs were seeded at a density of 5 × 10^3^ cells/cm^2^ (or 4.5 × 10^4^/well) in 6-well plates and cultured until fully confluent. Thereafter, a single microneedle device was applied onto the center of the well for a 12-h period. Microneedles were washed in phosphate buffered saline (PBS) and sterilized in 70% ethanol. Next, the microneedles were incubated in culture media (overnight) to pre-condition the microneedle devices prior to usage. Thus, the influence of potential soluble substances from LCP over the cells can be excluded. After treating KFs for 12 h, microneedles were removed, and fresh media containing Hoechst 33342 (NucBlue^®^, Life Technologies Singapore) and propidium iodide (PI, 10 μg/ml) was added, followed by incubation for 20 min. A control group was used for the imaging analysis that consisted of untreated cells that were almost confluent. An additional study group was evaluated in which microneedles were fixed to the wall of the well of cell culture plates. In this group, fibroblasts were allowed to grow at the bottom of the plate and care was taken to avoid direct contact of the cells with the microneedles. Cell culture medium was added to cover the entire microneedles device on the wall. This group allowed us to evaluate the effect of the material per cell on cell viability. The identical protocol was followed for the Hoechst 33342/PI labeling of all evaluated groups as well as for imaging. Thereafter, epi-fluorescence images (blue, red) and corresponding phase contrast images were obtained from the well center of control and microneedle-treated fibroblast cultures using the suitable filters and microscope settings. Images were acquired using 500 ms and 100 ms exposure time for red (PI) and blue (Hoechst 33342) fluorescence respectively using a LX71 inverted fluorescent (Olympus) microscope. The number of dead cells (PI-positive cells stained red) per view was counted and normalized per total number of cells (Hoechst 33342 positive cells stained blue) for *N* = 4. Cells were counted manually following staining of total cells (Hoechst 33342—blue) and non-viable cells (propidium iodide—red). After obtaining representative images for each experiment (*n* = 3), the fluorescent signal of both total and non-viable cells was tabulated to obtain the proportion of dead/total cells for untreated control and microneedle-treated groups. Approximately 386 ± 68.0 and 482 ± 132.8 cells per independent experiment for microneedle-treated and untreated control experiments were counted, respectively. This meant that the live/dead cell viability analysis was performed on >1000 cells per group.

### Rabbit ear hypertrophic scar

This model was adapted from a previous study [14]. Briefly, New Zealand white female rabbits were anesthetized using ketamine (60 mg/kg) and xylazine (5 mg/kg) before generating wounds on the rabbit ear. A total of 3 animal subjects were used, with 1 ear of each rabbit utilized. Each ear received 4 wounds with 2 serving as control wounds (mimicking hypertrophic scars) and 2 wounds which microneedles were applied. The microneedles were applied to the rabbit model throughout the experimental period without removal. The wounds were created using a 7-mm biopsy punch (Stiefel brand, Fisher Scientific Singapore), with flesh stripped down to bare cartilage on the ventral side of the ear. Care was taken to ensure the complete removal of epidermis, dermis, and perichondrium. Any signs of bleeding were treated with manual compression.

The wounds were either left to heal naturally (positive control) or a microneedle device applied (experimental group). For each rabbit ear, 4 wounds were randomly created with microneedle-treated and untreated wounds performed within the same ear to normalize inter-subject behavior (Fig. 3b). All wounds were fixed with 3M Tegaderm^®^ dressing and further secured using Matisol^®^ liquid adhesive. After 10 days, the rabbits were euthanized and their ears harvested for analysis (*N* = 3, both control and microneedle groups). Compassionate animal sacrifice was performed according to the institutional care and use committee (IACUC, #ARF-SBS/NIE-A0255) protocol of Nanyang Technological University, Singapore. The site of the original wound was harvested and stained with hematoxylin and eosin.

### Preliminary clinical procedure

**Fig. 1:**
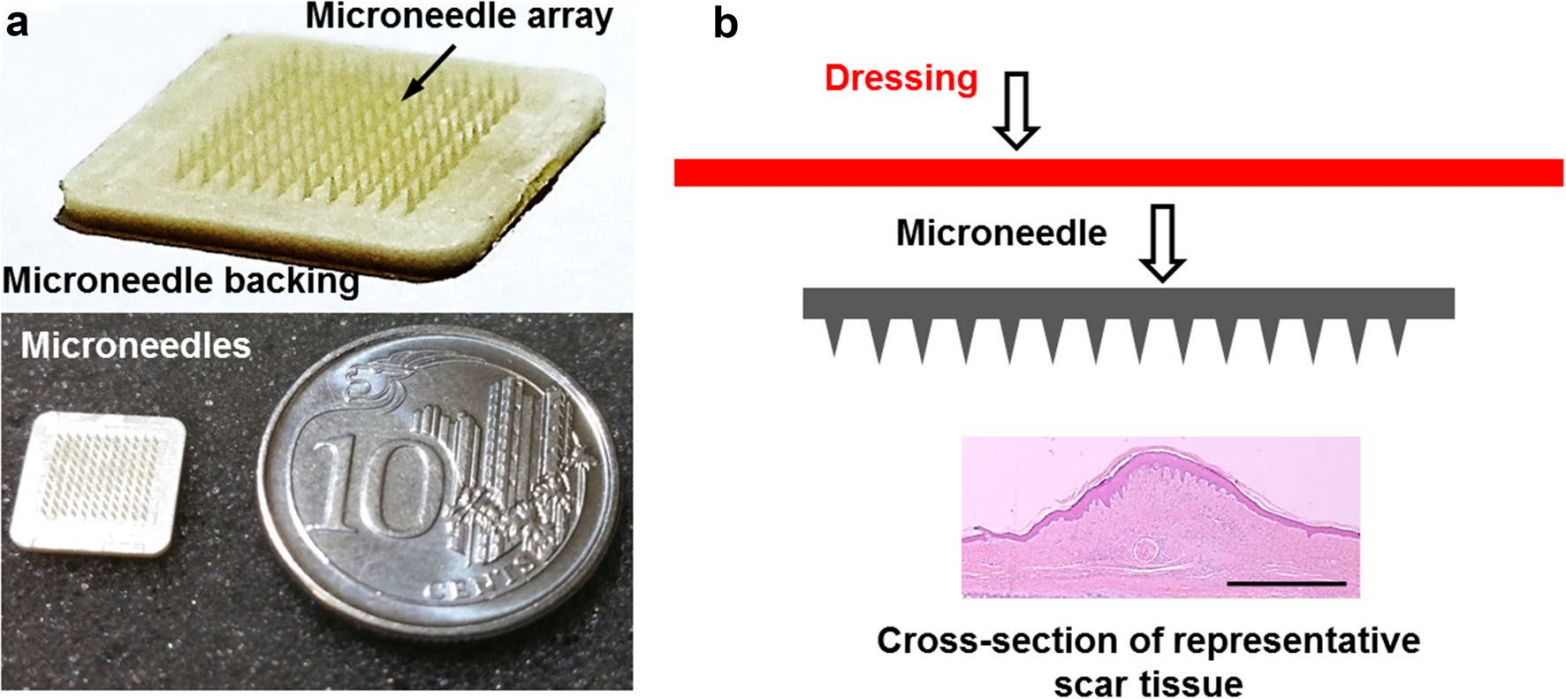
Drug-free microneedles for abnormal scar therapy. **a** A 10 × 10 array of microneedles (200 μm height) at 200 needle/cm density consisting of the array and its backing. Microneedle patch positioned adjacent to a coin of 1.85 cm diameter to illustrate device scale. **b** Microneedle administration scheme consists of placing the microneedle directly on the scar tissue and secured using dressing material (*figure not drawn to scale*). *Scale bar* represents 200 μm

The Microneedle patch was evaluated on a patient suffering from a single hypertrophic scar on the dorsum region following surgery. Consent was obtained from the patient for microneedle usage and the patient was treated until a significant reduction in inflammation (of the scar) could be observed. This was characterized by decreased redness, less itchiness experienced by the patient and the scar becoming less prominent. The treatment period lasted for 6 weeks before surgical scar revision was performed. The female patient had a single hypertrophic scar on her dorsum. She was young and healthy, with no known metabolic, endocrine or neoplastic diseases. Prior to applying microneedles, skin was cleaned and disinfected with 70% ethanol. The Microneedle device was positioned over the scar and fixed with translucent 3M Tegaderm^®^ tape. This is illustrated within the scheme in (Fig. 1). A total of 6 microneedle patches were applied lengthwise on the hypertrophic scar but a portion of it remained uncovered which served as the untreated control. The patient changed the microneedle patch every 2–3 days. After a significant reduction in scar inflammation was observed (decrease in redness and pruritus), the scar was completely excised to full thickness (epidermis and dermis) and analyzed following hematoxylin and eosin (H&E) and Masson–Goldner–Trichrome staining of histology sections.

### Histological assessment

Samples were collected and fixed with buffered formalin solution 10% for 24 h, and then transferred to 70% ethanol solution. Subsequently, the specimens were dehydrated and embedded in paraffin. 5-μm sections of each specimen were obtained and then stained with H&E and Masson–Goldner–Trichrome following standard protocols. The slides were observed and photographed with a microscope (Biorevo BZ9000, Keyence, Osaka, Japan) at different magnifications (e.g., ×4, ×10, ×20). A general picture of the entire histological section was performed using the software BZ-II Viewer and BZ-II Analyzer (Keyence, Osaka, Japan). For quantitative measurements of scar elevation, the scar elevation index measure was utilized. Please see [14] for the full details.

### Statistical analysis

Statistical analysis was carried out using either the student’s *t* test function from (Microsoft Excel, Singapore) with statistical significance determined at *p* < 0.05 or, One-way ANOVA and post hoc analysis for analysis containing 3 or more groups using the online ANOVA calculator available from http://www.astatsa.com with statistical significance determined at *p* < 0.01. Values are ≥3, and quoted as average ± standard deviation (SD).

## Results

Microneedle devices (with dimensions stated in the “Patients and methods” section) consist of an array of needles and its backing surface (Fig. 1a). Placing a single device adjacent to a coin (1.85 cm diameter) illustrates its relative size. To treat abnormal scars, microneedles were placed with the array portion contacting the epidermis, secured using suitable dressing materials (e.g., 3M Tegaderm^®^, Fig. 1b).

### Keloid fibroblast culture

**Fig. 2:**
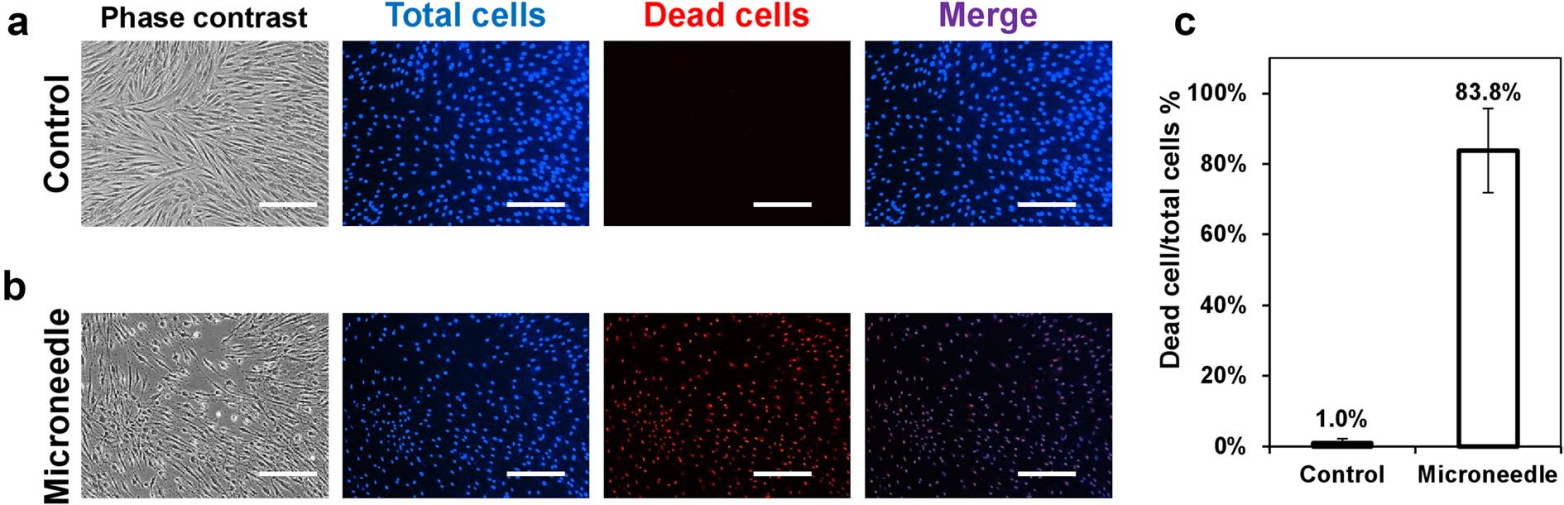
Microneedle application on keloid fibroblast culture. Representative phase contrast, *blue* and *red* fluorescence images of **a** control (untreated cells) and **b** microneedle-treated keloid cells incubated with Hoechst 33342 and propidium iodide. Images to the right are blue and red merged. **c** Quantification of dead/total cell proportion (%); *N* = 4, >1000 cells analyzed. *Scale bar* represents 100 μm

To assess the effect of microneedle treatment on keloids, an initial study was performed on keloid fibroblasts (KFs) that have a spindle-shaped morphology (Fig. 2a). Before treatment, both control and microneedle to-be-treated cells showed high cell density [(4.0 ± 1.11) × 10^5^ cells/cm^2^], indicated by Hoechst 33342 staining that labels both alive and dead cells. If cells were treated with microneedles for 12 h, a significant change in the morphology was observed (Fig. 2b). More impressively, the PI staining (PI only bound with DNA from dead cells that had ruptured nuclei) showed that 83.8 ± 11.96% of cells became non-viable. In contrast, there was no visible morphology change on the control or untreated cells. The proportion of dead cells was only 1.0 ± 1.13% of the total population (Fig. 2c), which was significantly smaller than the microneedle-treated group. This experiment demonstrates that physical contact (12 h) with drug-free microneedles causes KF cells to lose viability, suggesting that it is efficacious in preventing abnormal scar growth. Microneedle contact on normal fibroblast (NFs) cells similarly led to the loss of cell viability with a greater number of cells labeled with PI (Additional file 1: Figure S1A). It was also determined that microneedle contact, instead of material cytotoxicity, was responsible for inhibiting fibroblasts (Additional file 1: Figure S1B). Fibroblasts cultured in presence of the microneedles but avoiding direct physical contact with the needles showed normal growth with a negligible number of PI-positive cells. This observation goes in line with previous observations [11].

Further evaluation of the effect of microneedle contact on fibroblast cell death can be found in Additional file 1: Figure S2A, B. A significant increase on PI-positive cells was found on microneedle-treated cells when compared to untreated cells (*p* < 0.01). For this analysis, identical ROI (area were the needles were applied) was evaluated.

Interestingly, the inhibitory effect (measured by the proportion of cell death) was significantly higher for KF compared to NF cells (Additional file 1: Figure S1C).

### Rabbit ear hypertrophic scar model

**Fig. 3:**
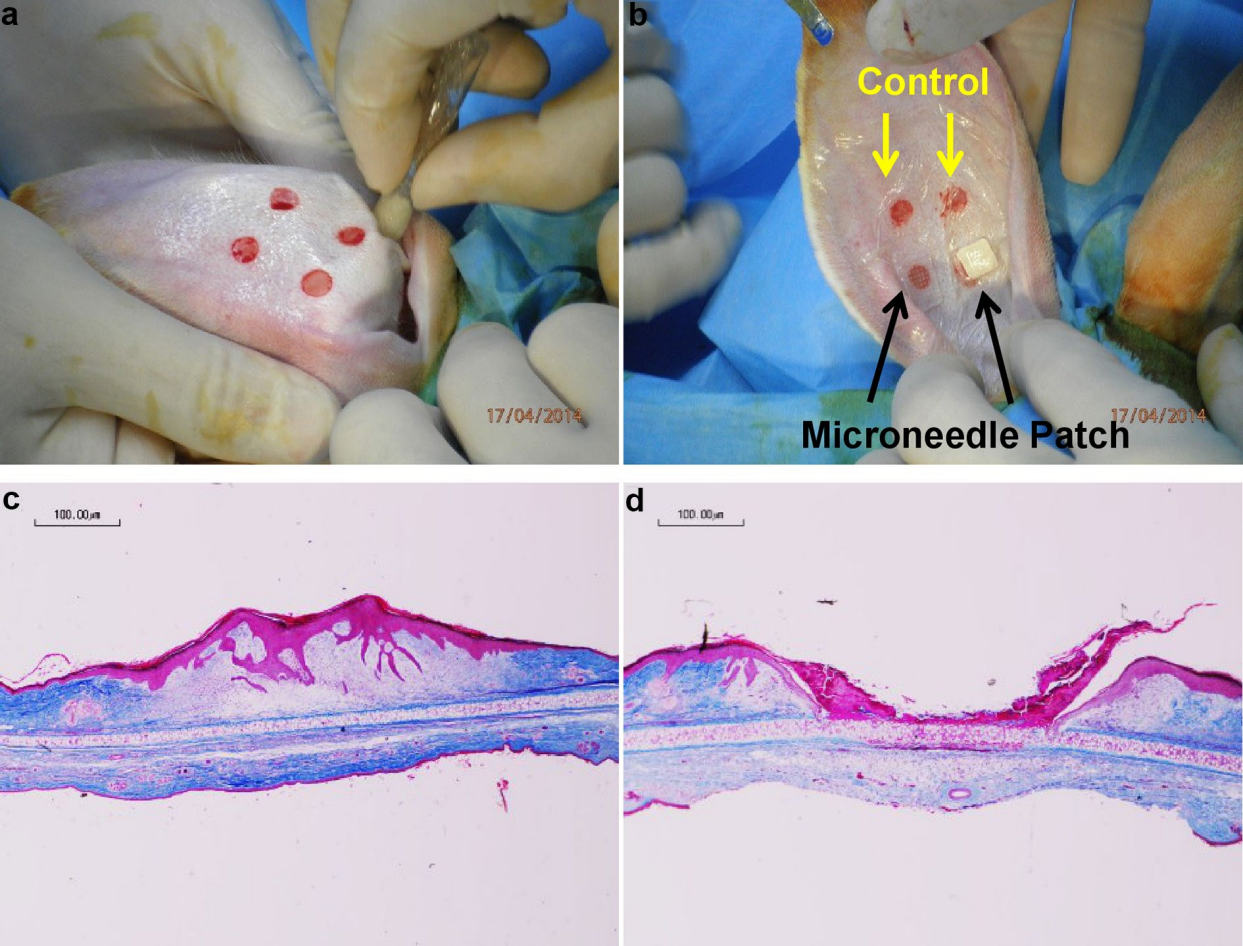
Microneedle application on rabbit ear hypertrophic scar model. **a** 4 wounds per rabbit ear were generated using a 7-mm punch biopsy. **b** Each rabbit ear had 2 wounds that were untreated (control) and 2 wounds treated (microneedle) before securing with wound dressing. **c** Representative cross-sectional images of control wounds after 10 days. **d** Representative cross-sectional images of microneedle-treated wounds after 10 days

We further assessed the efficacy of microneedle treatment on a rabbit ear hypertrophic scar model. Wounds created using punch biopsies on rabbit ears formed a region of raised tissue at the original site (Fig. 3a), which suitably modeled hypertrophic scars [14]. Then various treatments were applied on each wound (Fig. 3b). 10 days later, for control wounds, a raised region of tissue on the original wound site was observed (Fig. 3c) indicating the successful recapitulation of the hypertrophic scar model. On the other hand, microneedle application inhibited tissue growth and resulted a crater, preventing the tissue growth at the wound site (Fig. 3d). Quantification using the scar elevation index (SEI) measure (Additional file 1: Figure S3) showed a significant difference between the untreated wounds that developed a region of raised dermis (1.62 ± 0.14) and microneedle-treated wounds (0.80 ± 0.14) (Additional file 1: Figure S3A). Representative images of untreated and microneedle-treated wounds are shown to demonstrate how the SEI was obtained (Additional file 1: Figure S3B, C, respectively). All untreated wounds (100%) successfully formed raised tissue, whereas 66.7% of microneedle-treated wounds prevented its formation.

### Preliminary clinical experience

**Fig. 4:**
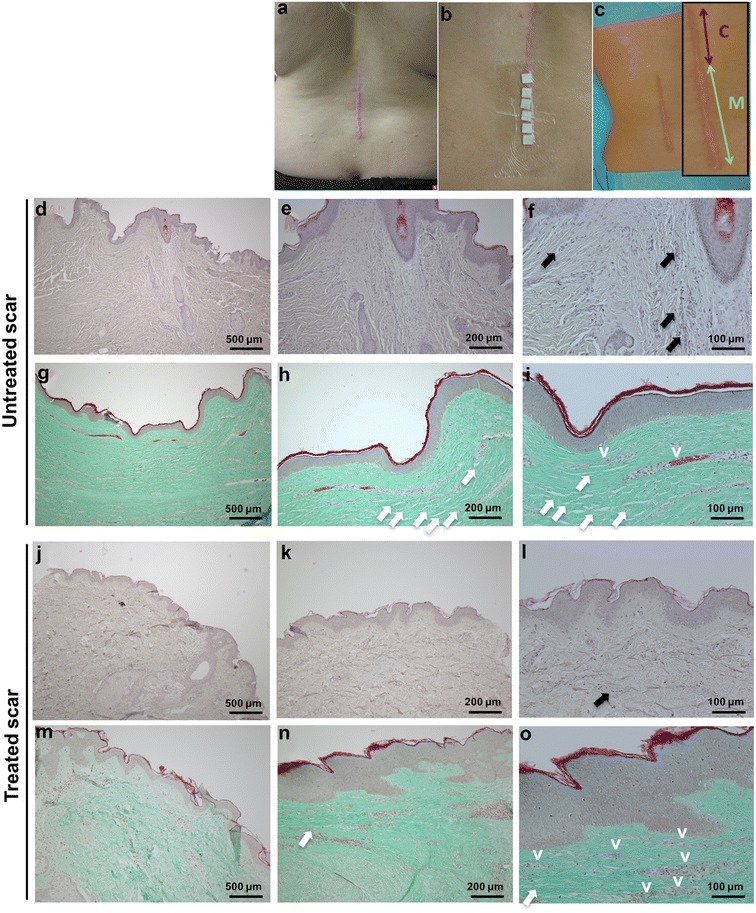
Microneedle patch application on a hypertrophic scar on the dorsal region. **a** Gross appearance of hypertrophic scar before treatment. **b** Application of the microneedle patches on the caudal half of the scar. **c** Hypertrophic scar following treatment (*inset* indicating control (*c*) and microneedle-treated (*M*) portions). Corresponding histology indicating a reduction of inflammatory signs compared to the untreated control. **d**–**f** H&E staining of the untreated control. Magnifications using ×4, ×10, and ×20, respectively. **g**–**i** Masson–Goldner–Trichrome staining of the untreated control. Magnifications using ×4, ×10, and ×20, respectively. **j**–**l** H&E staining of the treated scar. Magnifications using ×4, ×10, and ×20 respectively. **m**–**o** Masson–Goldner–Trichrome staining of the treated scar. Magnifications using ×4, ×10, and ×20, respectively. *Black arrows* indicate leukocyte infiltration. *White arrows* indicate disrupted connective tissue. *v* vessels

The patient observed a significant reduction in redness and pruritus after applying the microneedles. The patient was not disturbed in her daily activities wearing the microneedle patch. Histologic analysis was performed by means of H&E and Masson–Goldner–Trichrome staining. H&E allowed us to conclude on possible signs of tissue rejection to the patch application and/or inflammation (including infiltration of inflammatory cells). Masson–Goldner–Trichrome was used to evaluate connective tissue areas and architecture. The results indicated a significant reduction in chronic inflammation as it appeared that less leukocyte infiltration was observed (Fig. 4). A greater number of clinical subjects will be needed to validate this observation. Moreover, in the untreated scar tissue, dense fields of connective tissue without vascularization were observed (Fig. 4d–f). The dermal connective tissue had the appearance of a disrupted and disorganized architecture (Fig. 4g–i). On the other hand, the portion of the scar treated with microneedles showed a normal composition and dermal connective tissue architecture. Interestingly, no hyperdense connective tissue areas were observed (Fig. 4j–o) and almost no leukocytes were present.

## Discussion

To date, the main treatment options for abnormal scars—keloid and hypertrophic scars—are not considered satisfactory [15]. Briefly, the existing treatments can cause significant pain, are equipment-intensive and/or have inadequate efficacy. Herein, a superficially applied, drug-free microneedle device was utilized for treating abnormal (hypertrophic and keloid) scars.

Unlike many existing treatment options, microneedle treatment is based on physical contact. Hence its *modus operandi* implies that it can be categorized as a class I/II medical device. This eliminates reliance on the usage of toxic chemicals to inhibit abnormal scars, thereby mitigating against accompanying contraindications.

Results from cell culture, pre-clinical animal models, and limited clinical experiences are reported herein. A short period (12 h) of physical contact between microneedles and keloid fibroblasts resulted in significant loss of cell viability. Further studies indicated that the loss of cell viability on microneedle-treated samples was due to direct physical contact of the fibroblasts with the microneedles rather than as result of polymer material intrinsic toxicity. Extending this to pre-clinical models, microneedle contact inhibited the formation of raised dermis tissue on rabbit ear wound sites—an established model for hypertrophic scarring [14]. While human hypertrophic scars develop much later (3–6 months following the initial injury), the rabbit ear model was performed for a period of 10 days. This period was chosen because the model achieves maximum scar elevation 12 days post-injury. From our observation at day 18, the extent of scar elevation is significantly reduced. An interesting observation is the quality of treated scars which showed a ‘crater’ in the microneedle-treated wounds, possibly signifying overly potent inhibition of fibroblast migration/proliferation. Further studies are required to optimize the treatment protocol to facilitate adequate growth of dermal tissue which may be achieved by adjusting the frequency of microneedle application.

In a female human volunteer, microneedles were seemingly well tolerated with a significant reduction in ‘redness’ and pruritus. Reduced numbers of infiltrated leukocytes were also observed, with reduced disorganization seen in dermal tissue architecture. This suggests that drug-free microneedle devices are a promising drug-free, self-administered therapeutic for abnormal scars.

It was observed that drug-free microneedles caused the loss of cell viability following contact with keloid fibroblasts during in vitro culture through as-yet unknown mechanisms. Hypertrophic scars develop as a result of over-exuberant collagen synthesis and catabolism during the final stages of wound healing [16]. Likewise, we presume that microneedle contact interferes with this remodeling process to inhibit dermis tissue formation (rabbit ear model) and normalize connective tissue architecture (human patient) in a mature hypertrophic scar. Further studies will require a combination of in vitro and in vivo pre-clinical studies to validate the inhibitory effect of microneedle contact on collagen synthesis during the remodeling of wounds. It will be of great interest to further examine if the microneedle contact inhibition phenomenon is material dependent. Utilizing different microneedles and culture substrates comprising alternative materials will shed greater light as well as potentially identifying mechanism(s) on the manner in which microneedle contact inhibits abnormal scars. This may be due to different mechanical forces acting on the cells. Gap junction proteins (e.g., connexins) are a possible way in which microneedle contact inhibits abnormal scars. Further experiments to reveal/understand how microneedles interact with cells are currently being performed.

Herein, data were obtained only from a single patient. A larger patient cohort is required to provide stronger evidence of microneedle efficacy against abnormal scars. Further trials can even determine how application parameters determine microneedle efficacy. It would also be interesting to identify how microneedle parameters (density, height, thickness, etc.) modulate connective tissue architecture. Other areas to make improvements on include the following: examining how the period of microneedle application affects the scar as well as ensuring skin sterilization prior to microneedle application.

Microneedles discussed herein were fabricated from liquid crystalline polymer, which we chose for its favorable bio-safety properties, having attained prior FDA approval (Drug Master File No. 8468, Device Master File No. 315). A significant drawback is microneedle rigidity and inflexibility (182 MPa tensile stress @ break with Strain of 3.4%), which potentially impairs conformity to non-planar anatomical regions such as elbows and other joints. Liquid crystalline polymer can be substituted with more flexible biopolymers such as poly (ethylene glycol) diacrylate, which we have used previously [11] or even clinically compatible materials such as metal (titanium, stainless steel, nickel), silicon, and photolithographic epoxy [17].

## Conclusions

A self-administered drug-free microneedle device has been designed for treating abnormal scars. It inhibits scarring mechanisms through physical contact and it minimizes potential chemical side-effects that stem from drug-based therapeutics. Microneedle devices are cost-effective and can be self-administered at the user’s convenience. Positive results were demonstrated in this study using (i) in vitro cell culture (keloid fibroblast), (ii) in vivo animal model (rabbit ear scars), and (iii) evaluated on a single clinical subject. Although results indicate that microneedle application is efficacious, it needs to be verified on larger clinical cohorts. While it is imperative to promptly share these findings with the community, other concerns such as identifying the mechanism responsible for microneedle inhibition still exist. Thus, microneedle inhibition of abnormal scars is a highly promising therapeutic modality to treat abnormal scars.

## Additional file

**Additional file 1: Figure S1.** Microneedle contact and material cytotoxicity on fibroblasts. A) Representative phase contrast, blue and red fluorescence images of control and microneedle-treated normal fibroblasts (NF) incubated with Hoechst 33342 (blue) and propidium iodide (red). B) Fraction (%) of dead cells to total cells for the following studied groups: untreated control of normal fibroblasts (NF_C), normal fibroblasts cultured in presence of the microneedles but avoiding direct contact with the needles (NF_LCP) and microneedle-treated normal fibroblasts (NF_microneedle). The group NF_LCP aimed to evaluate the intrinsic toxicity of the material. For this, the microneedles were fixated to the walls of the well of the cell culture plate and immersed on the medium (schematically represented in B). C) Comparison of untreated control and microneedle-treated cells for keloids fibroblasts (KF) and normal fibroblasts (NF). Statistical significance has been indicated with *p < 0.01. **Figure S2.** Microneedles induce increased cell death. A) Representative images and B) quantification of dead Keloid Fibroblast (KF) cells per total cells in untreated controls and microneedle-treated cells. Quantification (B) was performed within the indicated (dotted lines) region of interest (ROI). The ROI was selected based on the region in which the microneedles were applied. This ROI from microneedle-treated samples was overlaid on the images of the untreated controls in order to have comparable ROI for quantification purposes. The used microscopic images were taken under identical fluorescence conditions and using the same magnification. Dead cells are propidium (PI—red)-labeled and total cells are Hoechst 33342 (blue)-labeled. Statistical significances are indicated with **p < 0.01, N = 3, values are mean ± SD. **Figure S3.** Quantified microneedle efficacy through the scar elevation index. A) SEI—scar elevation index measured on untreated & microneedle treated rabbit ear wounds. Representative untreated (B) and microneedle treated (C) wounds with the (**---**) region demarcating the raised neodermis and the (**―**) region signifying the original boundary where the wound was inflicted. *P < 0.01, N = 3.

## Abbreviations

FDA: US Food and Drug Administration
TAC: triamcinolone acetonide
LCP: liquid crystal polymer
DMEM: Dulbecco’s minimum essential medium
FBS: fetal bovine serum
PBS: phosphate buffered saline
PI: propidium iodide
H&E: hematoxylin and eosin
KF: keloid fibroblasts
NF: normal fibroblast
SEI: scar elevation index
