# Supplementary material for: Microneedle physical contact as a therapeutic for abnormal scars
Source: Eur J Med Res. 2017 Aug 14;22:28. doi: 10.1186/s40001-017-0269-6 (PMC5557429; doi:10.1186/s40001-017-0269-6)
Supplement: Supplementary file 1 — Additional file 1: Figure S1. Microneedle contact and material cytotoxicity on fibroblasts. A) Representative phase contrast, blue and red fluorescence images of control and microneedle-treated normal fibroblasts (NF) incubated with Hoechst 33342 (blue) and propidium iodide (red). B) Fraction (%) of dead cells to total cells for the following studied groups: untreated control of normal fibroblasts (NF_C), normal fibroblasts cultured in presence of the microneedles but avoiding direct contact with the needles (NF_LCP) and microneedle-treated normal fibroblasts (NF_microneedle). The group NF_LCP aimed to evaluate the intrinsic toxicity of the material. For this, the microneedles were fixated to the walls of the well of the cell culture plate and immersed on the medium (schematically represented in B). C) Comparison of untreated control and microneedle-treated cells for keloids fibroblasts (KF) and normal fibroblasts (NF). Statistical significance has been indicated with *p < 0.01. Figure S2. Microneedles induce increased cell death. A) Representative images and B) quantification of dead Keloid Fibroblast (KF) cells per total cells in untreated controls and microneedle-treated cells. Quantification (B) was performed within the indicated (dotted lines) region of interest (ROI). The ROI was selected based on the region in which the microneedles were applied. This ROI from microneedle-treated samples was overlaid on the images of the untreated controls in order to have comparable ROI for quantification purposes. The used microscopic images were taken under identical fluorescence conditions and using the same magnification. Dead cells are propidium (PI—red)-labeled and total cells are Hoechst 33342 (blue)-labeled. Statistical significances are indicated with **p < 0.01, N = 3, values are mean ± SD. Figure S3. Quantified microneedle efficacy through the scar elevation index. A) SEI—scar elevation index measured on untreated & microneedle treated rabbit ear wounds. Represent [file 40001_2017_269_MOESM1_ESM.docx]

Supplementary data

**Microneedle Physical Contact as a Therapeutic for Abnormal Scars**

Authors: David C Yeo^a^, Ph.D, Elizabeth R. Balmayor^b,c,d^, Ph.D, Jan-Thorsten Schantz ^a,b^, M.D., Ph.D, Chenjie Xu^a,e^, Ph.D.

**Affiliations:**

^a^ School of Chemical & Biomedical Engineering, Nanyang Technological University, Singapore

^b^ Department of Plastic Surgery and Hand Surgery, Klinikum Rechts der Isar, Technical University of Munich, Germany

^c^ Institute for Advanced Study, Technical University of Munich, Germany

^d^ Department of Experimental Trauma Surgery, Klinikum Rechts der Isar, Technical University of Munich, Germany

^e^ NTU-Northwestern Institute of Nanomedicine, Nanyang Technological University, Singapore

**Short running head** (≤40 characters): Microneedles for abnormal scars


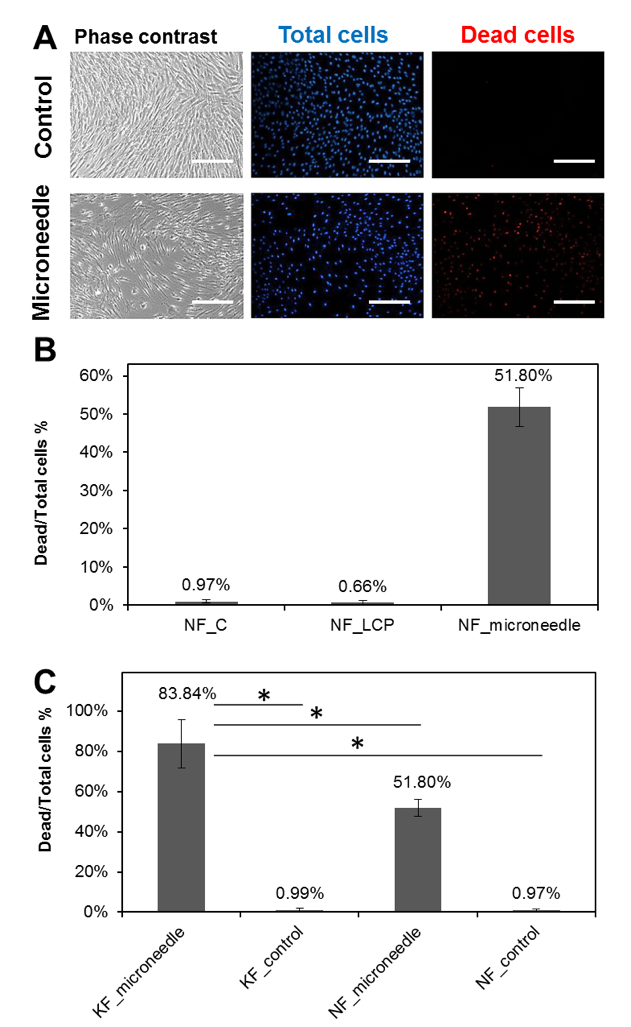

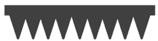

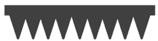


**Supporting Figure S1:** Microneedle contact and material cytotoxicity on fibroblasts. A) Representative phase contrast, blue and red fluorescence images of control and microneedle-treated normal fibroblasts (NF) incubated with Hoechst 33342 (blue) and propidium iodide (red). B) Fraction (%) of dead cells to total cells for the following studied groups: untreated control of normal fibroblasts (NF_C), normal fibroblasts cultured in presence of the microneedles but avoiding direct contact with the needles (NF_LCP) and microneedle-treated normal fibroblasts (NF_microneedle). The group NF_LCP aimed to evaluate the intrinsic toxicity of the material. For this, the microneedles were fixated to the walls of the well of the cell culture plate and immersed on the medium (schematically represented in B). C) Comparison of untreated control and microneedle-treated cells for keloids fibroblasts (KF) and normal fibroblasts (NF). Statistical significance has been indicated with *p<0.01.


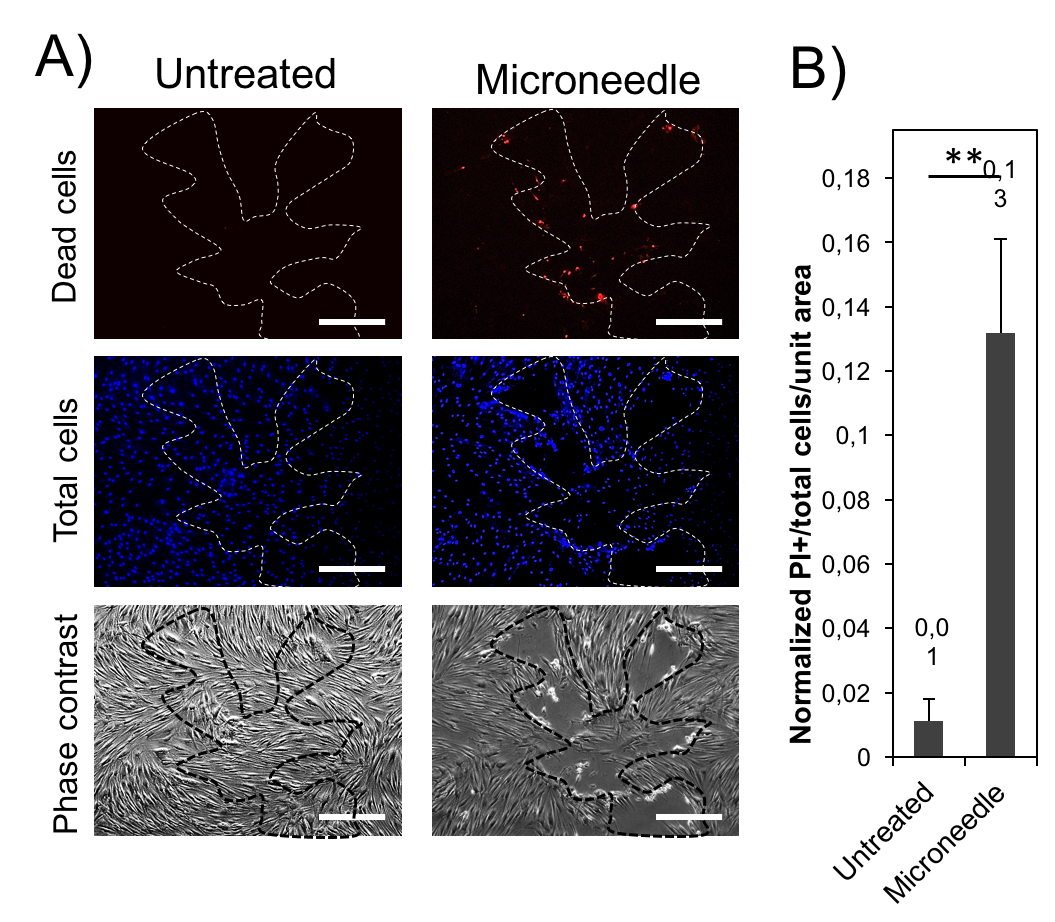
**Supporting Figure S2**: Microneedles induce increased cell death. A) Representative images and B) quantification of dead Keloid Fibroblast (KF) cells per total cells in untreated controls and microneedle-treated cells. Quantification (B) was performed within the indicated (dotted lines) region of interest (ROI). The ROI was selected based on the region in which the microneedles were applied. This ROI from microneedle-treated samples was overlaid on the images of the untreated controls in order to have comparable ROI for quantification purposes. The used microscopic images were taken under identical fluorescence conditions and using the same magnification. Dead cells are propidium (PI - red)-labeled and total cells are Hoechst 33342 (blue)-labeled. Statistical significances are indicated with **: p<0.01, N=3, values are mean±SD.


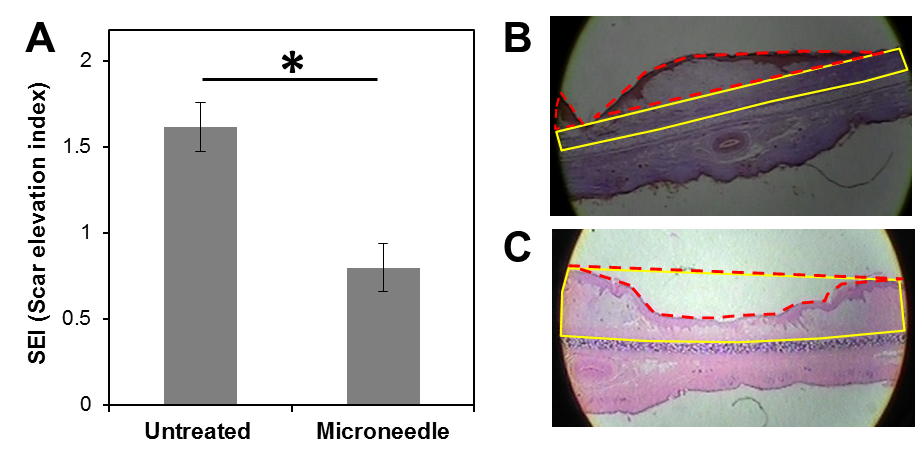


**Supporting Figure S3:** Quantified microneedle efficacy through the scar elevation index. A) SEI – scar elevation index measured on untreated & microneedle treated rabbit ear wounds. Representative untreated (B) and microneedle treated (C) wounds with the (**---**) region demarcating the raised neodermis and the (**―**) region signifying the original boundary where the wound was inflicted. *P<0.01, N=3.
